# Divergent clinical presentations and management of calcium-sensing receptor (CaSR) mutations: a case report

**DOI:** 10.1186/s13256-026-06062-3

**Published:** 2026-04-25

**Authors:** Stylianos Kopanos, Joachim Feldkamp

**Affiliations:** Academic Department of General Internal Medicine, Endocrinology, Diabetes and Infectiology, Bielefeld University, Medical School and University Medical Center East Westphalia-Lippe, Bielefeld, Germany

**Keywords:** Primary hyperparathyroidism (PHPT), Familial hypocalcuric hypercalcemia (FHH), Calcium-sensing receptor (CaSR) mutation, Parathyroid adenoma

## Abstract

**Background:**

Primary hyperparathyroidism (PHPT) and familial hypocalciuric hypercalcemia (FHH) are key differential diagnoses in parathyroid hormone (PTH)-mediated hypercalcemia. While PHPT often arises from single-gland parathyroid adenomas, CaSR mutations are typically associated with FHH. However, the coexistence of CaSR mutations and PHPT represents an unusual presentation, and the variability in their clinical impact remains underexplored. This report highlights two distinct cases of heterozygous CaSR mutations, including a novel mutation, shedding light on their potential roles in disease pathogenesis and management.

**Case presentation:**

The first case involves a 54-year-old Caucasian female with a heterozygous Ala986Ser CaSR mutation, PHPT due to a parathyroid adenoma, and autoimmune Graves’ disease. She presented with recurrent sicca syndrome, fatigue, hypercalcemia, elevated PTH, and hypercalciuria. Post-parathyroidectomy, persistent hypercalcemia and abnormal laboratory findings, alongside TRAK and TG antibodies, suggested a multifactorial pathogenesis. Imaging showed patchy hypoechoic thyroid parenchyma and recurrent adenoma. DXA revealed mild osteopenia, while calcimimetic therapy with cinacalcet was initiated but subsequently discontinued due to gastrointestinal intolerance. This unusual overlap of autoimmune and genetic factors emphasizes the complexity of managing PHPT with coexisting CaSR mutations.

The second case describes a 52-year-old Caucasian male with a heterozygous Glu1011Gln CaSR mutation. He presented with severe hypercalcemia, elevated PTH, nausea, and diffuse musculoskeletal pain. Imaging revealed no adenomas, but sonography later identified a hypoechoic lesion with central vascularization, suggestive of a potential adenoma. Initial symptomatic improvement occurred despite persistently elevated biochemical markers; however, clinical worsening with recurrent abdominal symptoms and progressive bone mineral density loss was observed during follow-up. This case highlights a possible association between CaSR variants and sporadic adenomas, underscoring diagnostic complexity rather than direct causality.

**Conclusions:**

These cases highlight the complex clinical presentations in patients carrying CaSR variants and autoimmune components, suggesting a broader spectrum of clinical phenotypes and pathogenesis than previously understood. The findings emphasize the importance of genetic analysis in atypical cases and underscore the need for further research into the role of CaSR mutations in PHPT, which may inform future diagnostic and therapeutic strategies.

**Supplementary Information:**

The online version contains supplementary material available at 10.1186/s13256-026-06062-3.

## Introduction

Primary hyperparathyroidism (PHPT) is the leading cause of hypercalcemia, primarily due to a single parathyroid adenoma. While asymptomatic hypercalcemia is the most common presentation, PHPT can lead to complications, such as bone demineralization and kidney damage, including nephrolithiasis and nephrocalcinosis [1].

PHPT is one of the most prevalent endocrine disorders, with an estimated prevalence of 1–7 cases per 1000 adults. Accurate incidence determination is challenging due to variations in global screening practices, case definitions, study populations, and annual fluctuations. In 80–90% of cases, PHPT is caused by a single parathyroid adenoma, with four-gland hyperplasia accounting for 10–15%, multiple adenomas for 5%, and parathyroid malignancies for less than 1%. It can also occur in familial endocrine syndromes, such as multiple endocrine neoplasia (MEN) types 1 and 2A, as well as isolated familial hyperparathyroidism [2, 3].

Familial hypocalciuric hypercalcemia (FHH) is a rare, benign genetic disorder marked by mild to moderate hypercalcemia, low urinary calcium excretion, and parathyroid hormone (PTH) levels that are normal or slightly elevated. It is often asymptomatic and typically identified incidentally during routine bloodwork. While FHH can occur at any age, severe cases are most commonly seen in infancy [4].

FHH is usually inherited in an autosomal dominant manner with high penetrance, meaning most individuals with the mutation display symptoms, though the severity may vary (variable expressivity) [5]. The condition is frequently caused by heterozygous loss-of-function mutations in the *CaSR* gene, which encodes the calcium-sensing receptor (CaSR). This G-protein-coupled receptor is essential for calcium regulation, playing a pivotal role in controlling PTH secretion and calcium excretion in the kidneys [6, 7].

CaSR, part of the family C G-protein-coupled receptors, detects extracellular calcium (Ca^2^⁺), magnesium (Mg^2^⁺), amino acids, peptides, anions, and pH changes, influencing cellular functions, such as gene expression, cell proliferation, differentiation, apoptosis, muscle contraction, and neuronal activity. Dysfunction of CaSR is linked to conditions, such as autosomal dominant hypocalcemia, FHH, and neonatal severe hyperparathyroidism [8].

Recent studies have expanded knowledge of CaSR’s structural and biochemical properties, including its binding sites for natural ligands and modulators. Over 400 *CaSR* mutations have been identified, impacting processes, such as protein trafficking, surface expression, endocytosis, degradation, and signalling, shedding light on the molecular basis of CaSR-related disorders [8–10].

**Fig. 1:**
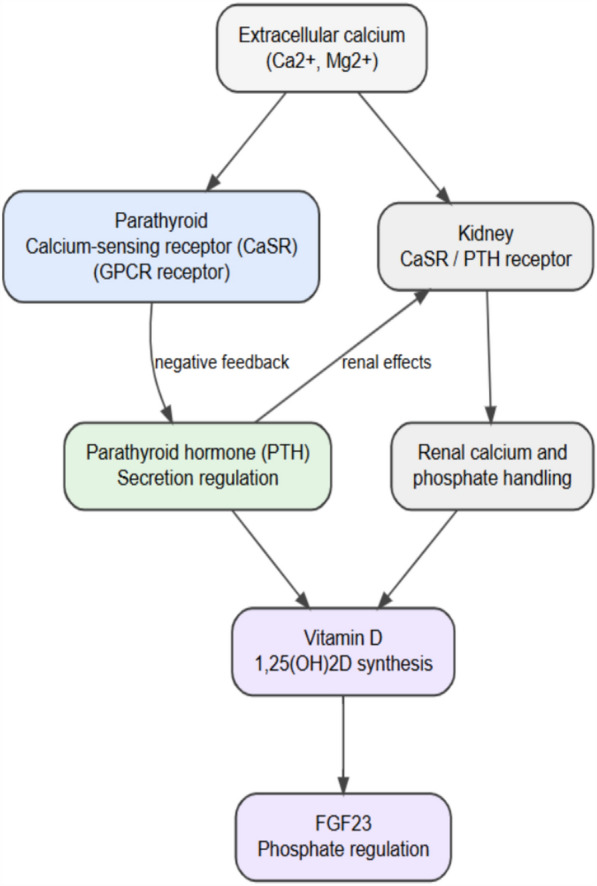
Schematic illustration of key molecular pathways involved in calcium–phosphate homeostasis and calcium-sensing receptor (CaSR)-mediated signaling. The figure summarizes established physiological mechanisms involving CaSR, parathyroid hormone (PTH), vitamin D, and fibroblast growth factor 23 (FGF23), based on concepts described in the literature

CaSR is primarily expressed in the parathyroid glands and kidneys. Normally, low serum calcium levels activate CaSR to stimulate PTH secretion and enhance renal calcium reabsorption. Mutations in the *CaSR* gene decrease the receptor's calcium sensitivity, leading to elevated serum calcium levels required to suppress PTH secretion. In the kidneys, these mutations increase calcium and magnesium reabsorption, resulting in hypercalcemia, hypocalciuria, and often high-normal magnesium levels. For context, the key physiological pathways linking CaSR signaling, PTH regulation, renal calcium handling, vitamin D, and phosphate balance are summarized in Fig. 1.

Although inactivating *CaSR* mutations are not strongly associated with parathyroid tumors in sporadic primary hyperparathyroidism (PHPT), decreased CaSR expression is frequently observed in hyperplastic and adenomatous parathyroid tumors. A notable case involved the coexistence of an inactivating *CaSR* mutation with PHPT linked to a single parathyroid adenoma [11–13].

FHH causes lifelong hypercalcemia, which is generally asymptomatic. When symptoms occur, they are usually mild, including fatigue, weakness, and cognitive disturbances. Physical appearance remains unaffected, and there is no increased risk of urolithiasis or osteopenia, although rare cases of chondrocalcinosis and acute pancreatitis have been documented.

Diagnosing FHH can be challenging due to biochemical variability and similarities with other hypercalcemia causes, such as primary hyperparathyroidism. Genetic testing is often useful in distinguishing FHH from other conditions [14].

Management primarily involves regular monitoring, given the typically benign nature of the condition, which rarely requires treatment. Unnecessary surgical procedures should be avoided. Genetic counselling is recommended for affected families to provide reassurance and education about the condition’s benign nature. Calcimimetics, which are allosteric activators of CaSR, may offer therapeutic potential for conditions involving hypoactive CaSR, such as FHH [15].

According to local institutional regulations, formal ethical approval was not required for anonymized single case reports. Written informed consent for publication was obtained from both patients.

## Case 1

### Case presentation

A 54-year-old Caucasian origin female was referred for the management of iron deficiency anemia. Her medical history included endometriosis, uterine myomas, and autoimmune thyroid disease (Graves' disease). Endoscopic evaluations did not identify a bleeding source. During her hospital stay, she experienced an allergic reaction to a transfusion, marked by fever and elevated inflammatory markers. She also reported a history of hypercalcemia, identified in early adulthood, which led to the discovery of a right parathyroid adenoma with accompanying left hyperplasia. Both were surgically removed without complications, and histopathology revealed no malignancy. A family history of hypercalcemia was noted among her cousins. Additional symptoms included neck swelling, weight gain, Sicca syndrome, breast tenderness, libido loss, anxiety, and depression. Her only medication was iron supplements, and physical examination was significant for an elevated BMI but otherwise unremarkable findings.

### Investigation

Post-parathyroidectomy laboratory tests revealed corrected calcium of 2.35 mmol/L (reference range: 2.2–2.55 mmol/L), PTH of 65.3 pg/mL (reference range: 15–65 pg/mL), phosphorus of 3.3 mg/dL (reference range: 2.7–4.5 mg/dL), and vitamin D levels of 14.7 ng/mL (reference range: > 20 ng/mL). A 24-h urine calcium level was elevated at 12.7 mmol/24 h (reference range: 2.5–8 mmol/24 h). Hemoglobin, initially 4.6 g/dL (reference range: 11.6–15 g/dL), improved to 9.5–10 g/dL following transfusion. Ferritin was < 10 ng/mL (reference range: 15–250 ng/mL). Preoperative PTH levels were 274 pg/mL.

Neck ultrasound showed transient hypervascularization of the thyroid gland, consistent with elevated TRAK levels (3.53 U/L; reference range: 0–1.75 U/L), supporting a diagnosis of Graves' disease. Subsequent symptoms of numbness, musculoskeletal pain, fatigue, heat intolerance, and hot flashes were attributed to perimenopause. Due to weight gain, the patient was started on levothyroxine 50 μg. Lumbar MRI revealed recurrent osteochondrosis.

Five years later, she presented with biochemical findings suggestive of recurrent parathyroid dysfunction, including borderline hypercalcemia (2.5 mmol/L), elevated PTH levels (74.9 pg/L) and vitamin D deficiency (14.7 ng/mL). Neck ultrasound showed patchy hypoechoic thyroid parenchyma, and dual-energy X-ray absorptiometry DEXA revealed a 4% cumulative bone density loss, consistent with osteopenia. Initially, no parathyroid adenomas were initially detected, and renal ultrasound ruled out nephrolithiasis. Given her family history and recurrent hyperparathyroidism, genetic testing identified a heterozygous CaSR variant, p.(Ala986Ser) (ClinVar Variation ID: 2140), a common variant that has been variably reported as a polymorphism with weak or uncertain pathogenicity.

The *Ala986Ser* variant, involving the substitution of alanine with serine at position 986 within the intracellular domain of the *CaSR*, is associated with altered calcium regulation. While this variant does not form abnormal intermolecular bonds, it is hypothesized to affect intracellular signalling pathways, contributing to mild dysregulation of calcium homeostasis. The *Ala986Ser* variant is more common than other pathogenic *CaSR* mutations and is often considered a polymorphism. It is associated with primary hyperparathyroidism (PHPT) and subtle calcium-regulatory changes. According to gnomAD, its allele frequency is approximately 10.7%. ClinVar classifies the variant as relatively benign.

### Treatment

Initial treatment with cinacalcet was discontinued by the patient due to gastrointestinal side effects. Vitamin D supplementation was maintained; however, PTH levels remained elevated (108.2 pg/L), with persistently low vitamin D levels (19.3 ng/mL). Subsequent neck ultrasound identified an 8.1 mm epithelial adenoma, raising the question of surgical revision. Follow-up care was discontinued due to the patient’s non-attendance.

## Case 2

### Case presentation

A 52-year-old Caucasian origin male presented with persistent unexplained hypercalcemia and markedly elevated parathyroid hormone (PTH) levels. Symptoms included vertigo, recurrent myalgia, arthralgia, polyuria, and haematuria, while nausea, polydipsia, headache, constipation, and personality changes were absent. Imaging studies, including neck ultrasound, sestamibi scan, and MRI of the neck and pituitary with contrast, revealed no parathyroid or thyroid abnormalities. Dual-energy X-ray absorptiometry (DEXA) showed no signs of osteopenia, and renal ultrasound was negative for calculi.

The patient’s medical history included hypertension, dyslipidaemia, and a transient ischemic attack (TIA) with no residual symptoms. He had no significant surgical history. Current medications included aspirin, simvastatin, and candesartan. There was no family history of renal insufficiency or urolithiasis.

### Investigation

Laboratory investigations confirmed hypercalcemia with corrected calcium levels of 2.9 mmol/L (reference range: 2.15–2.5 mmol/L) and elevated PTH levels of 72.4 pg/L (reference range: 15–65 pg/L). Vitamin D levels were within the normal range at 40.7 ng/mL (> 20 ng/mL). A 24-h urine analysis showed calcium and phosphorus levels at the upper-normal or slightly elevated range (calcium: 8.6 mmol/24 h, reference range: 2.5–8 mmol/24 h; phosphorus: 1.26 mmol/24 h, reference range: 0.34–1 mmol/24 h) with a urine volume of 2350 mL. Pituitary MRI with contrast was performed as part of an extended endocrine work-up to exclude syndromic or central causes of PTH dysregulation, despite the absence of overt clinical features suggestive of MEN.

Genetic testing for *AP2S1, CASR, GNA11, CDKN1B-2B/C,* as well as *MEN1* and *RET* proto-oncogenes, identified a rare missense variant in the *CASR* gene (p.(Glu1011Gln)). At the time of analysis, this variant was not listed in the ClinVar database. In silico analysis suggested that this intracellular-domain variant may alter CaSR function through effects on receptor trafficking and/or intracellular signaling coupling. Given the location within the cytoplasmic tail, a plausible mechanism includes impaired membrane expression, altered interactions with regulatory proteins, or modified downstream signaling efficiency. No functional in vitro validation was performed, and mechanistic inferences, therefore, remain hypothetical.

The variant, with an allele frequency of 0.000008 in the general population (gnomAD), involves the substitution of glutamic acid with glutamine at position 1011 of the CaSR. It has been associated with familial hypocalciuric hypercalcemia (FHH) and neonatal severe hyperparathyroidism (NSHPT). Conservation analysis suggests that residues in functional domains, such as the intracellular region of CaSR, are highly conserved, supporting the pathogenicity of this mutation. Despite genetic counselling being recommended, relatives declined genetic testing.

### Follow-up

A conservative “watch-and-wait” strategy was adopted. Initial calcium and PTH levels remained stable, although 25-hydroxyvitamin D levels decreased. This approach was chosen, because initial imaging studies did not clearly identify a parathyroid adenoma and the presence of a CaSR variant introduced diagnostic uncertainty regarding the differentiation between primary hyperparathyroidism and familial hypocalciuric hypercalcemia. In this context, a period of biochemical surveillance was considered clinically appropriate before proceeding to potential surgical intervention.

Over time, the patient reported increasing abdominal pain and nausea during routine endocrinological surveillance. DEXA revealed a progressive trend toward osteopenia, with an average 5.8% reduction in bone mineral density. Neck ultrasound subsequently identified a 5 mm parathyroid adenoma. The patient was started on vitamin D supplementation (1000 IU daily) to address the documented insufficiency.

**Fig. 2:**
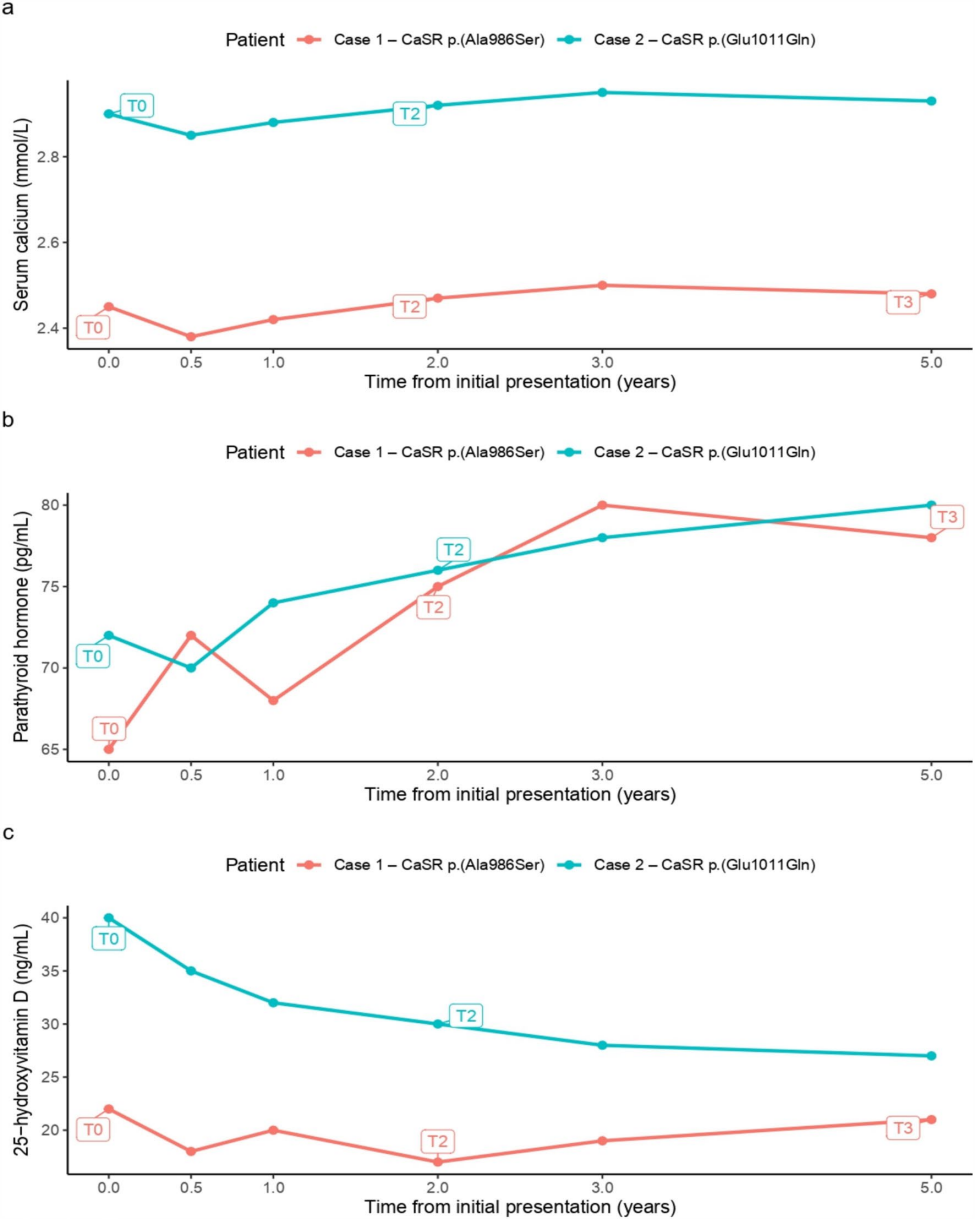
**a**–**c** Longitudinal biochemical trends in Case 1 and Case 2. Serum calcium, parathyroid hormone (PTH), and 25-hydroxyvitamin D levels are shown over time relative to initial presentation (T0). Data points represent clinically obtained measurements during routine follow-up. Case 1 corresponds to CaSR p.(Ala986Ser), and Case 2 corresponds to CaSR p.(Glu1011Gln). **T0** = initial presentation. **T1** = post-surgery (Case 1)/initial evaluation (Case 2). **T2** = follow-up. **T3** = recurrence/symptom progression

To illustrate the evolving biochemical course and its relationship to symptoms and management decisions, longitudinal trends in serum calcium, PTH, and 25-hydroxyvitamin D are presented in Fig. 2.

## Discussion

To this point, mutations in the *CaSR* gene have been associated with familial hypocalciuric hypercalcemia (FHH), the majority of which are loss-of-function mutations, encompassing missense, nonsense, and frameshift alterations. These mutations vary in their impact on receptor function, leading to diverse clinical outcomes. As highlighted by Lee et al., this genetic and clinical heterogeneity underscores the complex presentation of *CaSR*-related disorders [17].

In this report, Case 1 involved the *CaSR* p.(Ala986Ser) variant, which is associated with subtle calcium regulation abnormalities and possible recurrence of parathyroid dysfunction, illustrating how even benign polymorphisms may contribute to clinical variability. Conversely, Case 2 presented a rare *CaSR* p.(Glu1011Gln) mutation, absent in population databases and the literature, resulting in severe calcium homeostasis disruptions consistent with primary hyperparathyroidism (PHPT). This mutation demonstrates the significant pathogenic potential of rare *CaSR* variants.

The CaSR p.(Ala986Ser) variant is among the most frequently reported CaSR variants in the general population and is widely considered a common polymorphism with weak or absent pathogenicity. Its clinical significance remains controversial, as it has been variably associated with subtle alterations in calcium–parathyroid hormone homeostasis but is not regarded as a direct cause of primary hyperparathyroidism. In this context, the presence of the Ala986Ser variant in Case 1 should be interpreted with caution. Rather than serving as a causal pathogenic driver, it may represent a phenotype modifier that contributes to biochemical variability and diagnostic complexity, particularly when coexisting with other endocrine conditions and fluctuating vitamin D status. A structured comparison of the two CaSR variants (p.(Ala986Ser) and p.(Glu1011Gln)) and their contrasting clinical implications is provided in Table 1. This comparison highlights the differing levels of evidence supporting variant interpretation and reinforces the need for cautious genotype–phenotype attribution.

**Table 1:** Comparison and contrast of the Glu1011Gln and Ala986Ser mutations in the *CaSR* gene

Feature	Glu1011Gln mutation	Ala986Ser mutation
Position	Amino acid position 1011	Amino acid position 986
Substitution	Glutamic acid (Glu) → Glutamine (Gln)	Alanine (Ala) → Serine (Ser)
Domain affected	Intracellular domain	Intracellular domain
Functional impact	– Predicted to alter intracellular signaling and/or receptor trafficking; functional validation not performed – Impairs calcium-sensing activity	– Does not form disulfide bonds – Hypothesized to affect intracellular signalling pathways, leading to subtle dysregulation of calcium homeostasis
Association with conditions	– Familial hypocalciuric hypercalcemia (FHH) – Neonatal severe hyperparathyroidism (NSHPT)	– Primary hyperparathyroidism (PHPT) – Minor alterations in calcium regulation
Pathogenicity	Strongly pathogenic	Generally considered a polymorphism with weaker pathogenic potential
Frequency in population	Extremely rare (allele frequency: 0.000008 in gnomAD)	More common (allele frequency: ~ 10.7% in gnomAD)
ClinVar classification	Pathogenic	Relatively benign
Mechanistic insight	Potential impact on intracellular CaSR signaling or receptor trafficking; functional consequences remain unvalidated	Alters intracellular signalling pathways, potentially modifying calcium regulation without major structural disruptions
Severity of effects	Associated with significant disruption of calcium regulation, often with clinical symptoms	Mild effects, often asymptomatic or with minor clinical manifestations
Treatment implications	Genetic counselling and regular monitoring; potential for calcimimetics	Typically no specific treatment required; may influence mild PHPT management

In Case 1, persistent vitamin D deficiency during follow-up represents an important confounding factor when interpreting elevated parathyroid hormone levels. Vitamin D deficiency can contribute to secondary hyperparathyroidism and may have partially influenced the biochemical profile observed in this patient. Therefore, the interpretation of recurrent primary hyperparathyroidism should be approached with caution, and a multifactorial explanation including vitamin D deficiency, previous parathyroid disease, and possible CaSR-related modulation of calcium homeostasis is likely.

Urinary calcium excretion further illustrates the diagnostic ambiguity observed in the present cases. In Case 1, documented hypercalciuria is not consistent with classic familial hypocalciuric hypercalcemia and argues against CaSR loss-of-function-mediated hypocalciuria, supporting a phenotype more compatible with primary hyperparathyroidism or secondary influences on calcium metabolism. Because the patient was subsequently lost to follow-up, repeat urinary calcium measurements after correction of vitamin D deficiency could not be performed. This represents a limitation when attempting to definitively differentiate between recurrent primary hyperparathyroidism and other calcium-regulatory disturbances. In contrast, Case 2 demonstrated urinary calcium values at the borderline of the normal range, which neither clearly support nor exclude CaSR-related hypocalciuric physiology. This intermediate biochemical profile contributed to diagnostic uncertainty and complicated differentiation between PHPT and FHH. Together, these findings highlight that urinary calcium indices may vary over time and across clinical contexts, reinforcing the importance of longitudinal evaluation rather than reliance on single biochemical thresholds.

While hypocalciuria is often regarded as a diagnostic feature of FHH, as emphasized by Vannucci et al., the cases here showed deviations, with hypercalciuria in Case 1 and normocalciuria in Case 2. In addition, the overlap of symptoms with other conditions, such as PHPT and Graves' disease, as seen in Case 1, further complicates diagnosis and underscores the diverse clinical manifestations of *CaSR* mutations, irrespective of their molecular profiles [18]. Accordingly, we have limited mechanistic interpretation of p.(Glu1011Gln) to plausible intracellular-domain effects and explicitly acknowledge the absence of functional confirmation.

Gorvin et al. discuss potential molecular pathways of *CaSR* mutations, including their roles in parathyroid cell proliferation, hyperplasia, and tumour formation, though conclusions remain uncertain. In both cases, the association between *CaSR* mutations and parathyroid adenomas warrants further exploration [13, 19, 20]. The occurrence of PHPT alongside a *CaSR* mutation is exceedingly rare, and this report contributes such a case to the literature. The possibility of a causal link between *CaSR* mutations and parathyroid tumors highlights the need for broader molecular and histopathological studies.

Reduced surface expression of the *CaSR* protein, as seen in parathyroid adenomas, has been associated with impaired calcium-mediated suppression of parathyroid hormone (PTH) release. This impaired regulation may contribute to the pathophysiology of PHPT in the presence of *CaSR* mutations. However, current data on the prevalence of *CaSR* mutations in PHPT remains limited. Documented inactivating *CaSR* mutations have been observed in familial benign hypercalcemia, neonatal severe hyperparathyroidism, FHH, and Bartter syndrome, but their role in PHPT requires further study [21].

Regarding management, the conservative "watch-and-wait" approach in Case 2, supplemented with vitamin D, was effective until adenoma recurrence. In contrast, cinacalcet in Case 1 was poorly tolerated, highlighting the need for individualized therapy and regular monitoring. Calcimimetic agents, such as cinacalcet, enhance *CaSR* affinity for calcium and reduce PTH secretion, as emphasized by Tian et al. [22]. While approved for tertiary hyperparathyroidism, parathyroid carcinoma, and PHPT in specific scenarios, calcimimetics have also shown potential in managing symptomatic FHH and neonatal severe hyperparathyroidism due to *CASR* and *AP2S1* mutations. Controlled trials are needed to evaluate their broader utility for hypercalcemia unresponsive to conservative measures.

Limitations of this report include the absence of long-term follow-up for both cases, hindering a comprehensive understanding of disease progression. In addition, the lack of genetic testing among Case 2's relatives limits insights into familial implications. Genetic counselling is essential to elucidate inheritance patterns and inform management strategies. The calcium/creatinine clearance ratio (CCCR) is commonly used to aid differentiation between primary hyperparathyroidism and familial hypocalciuric hypercalcemia. However, its interpretation may be limited in complex or evolving clinical scenarios. In the present cases, CCCR could not be consistently calculated at all timepoints due to the lack of contemporaneous serum and urinary measurements, as well as fluctuations in calcium levels and vitamin D status over time. This limitation contributed to diagnostic uncertainty and illustrates a common real-world challenge, where reliance on a single CCCR measurement may be insufficient. Longitudinal assessment integrating biochemical trends, imaging findings, and clinical evolution was, therefore, required to guide management decisions. Furthermore, the absence of systematic family phenotyping and segregation data is also important. Although Case 1 reported a family history of hypercalcemia, contemporaneous biochemical results from relatives were not available. In Case 2, relatives declined further evaluation. Therefore, segregation of the identified CaSR variants with biochemical phenotypes could not be assessed, and no causal inference can be made on this basis.

In both cases, the limitations of biochemical markers and imaging had direct implications for clinical decision-making. Single-point measurements of serum calcium, parathyroid hormone, or urinary calcium excretion were insufficient to clearly distinguish between primary hyperparathyroidism and CaSR-related phenotypes, particularly in the presence of confounding factors such as vitamin D deficiency and evolving disease course.

Similarly, inconclusive or delayed imaging findings contributed to uncertainty regarding the timing of surgical intervention versus conservative management. These diagnostic ambiguities necessitated a cautious, longitudinal approach, highlighting the importance of integrating serial biochemical trends, imaging over time, and clinical symptom progression when formulating individualized management strategies.

Taken together, our findings support the concept that CaSR variants should be interpreted as potential phenotype modifiers that influence biochemical variability and diagnostic uncertainty, rather than as primary causative factors of parathyroid tumorigenesis. These cases illustrate the diagnostic complexity that may arise when genetic findings coexist with biochemical and imaging features that do not fully conform to classical diagnostic patterns. In such situations, clinical decision-making often requires longitudinal assessment rather than reliance on single biochemical measurements or genetic findings alone. The integration of clinical presentation, biochemical trends, imaging results, and genetic data remains essential for appropriate management of patients with suspected disorders of calcium homeostasis.

The availability of ClinVar annotations further underscores the contrasting levels of evidence supporting the clinical interpretation of common versus rare CaSR variants. Further research is necessary to explore the intracellular signalling pathways altered by *Ala986Ser* and its molecular mechanisms. In addition, large-scale studies on rare *CaSR* mutations, such as *Glu1011Gln*, are critical to understanding their clinical variability and improving therapeutic strategies. Importantly, genetic findings should be interpreted as complementary to, rather than replacements for, established biochemical and clinical criteria guiding management decisions in primary hyperparathyroidism.

## Conclusion

This case report highlights that CaSR variants may be associated with divergent clinical phenotypes and diagnostic complexity rather than acting as direct pathogenic drivers of primary hyperparathyroidism. The presented cases illustrate how CaSR variants can modify calcium–parathyroid hormone homeostasis and complicate the differentiation between primary hyperparathyroidism and familial hypocalciuric hypercalcemia in real-world clinical practice.

These observations underscore the importance of cautious interpretation of genetic findings, integration of longitudinal biochemical data, and individualized clinical decision-making, further highlighting that diagnostic tools, such as CCCR, while useful, should be interpreted within a longitudinal and clinical context rather than as isolated determinants of diagnosis. Advances in tools for analysing rapid calcium dynamics can enhance understanding of CaSR-mediated signalling and accelerate drug discovery. Emphasis should be placed on individualized treatment plans, regular monitoring, and patient education to achieve optimal outcomes.

## Supplementary Information

Additional file1 (PDF 287 kb)

## Data Availability

The data supporting the findings of this study are stored locally at Klinikum Bielefeld, Germany, and are available from the corresponding author upon reasonable request.
