# Supplementary material for: Divergent clinical presentations and management of calcium-sensing receptor (CaSR) mutations: a case report
Source: J Med Case Rep. 2026 Apr 25;20:221. doi: 10.1186/s13256-026-06062-3 (PMC13109886; doi:10.1186/s13256-026-06062-3)
Supplement: Supplementary file 1 — Additional file1 (PDF 287 kb) [file 13256_2026_6062_MOESM1_ESM.pdf]

CARE case report guidelines

CARE Checklist of information to include when writing a case report

(cc) BY-NC-ND

Topic	Item	Checklist item description	Reported on Line
Title	1	The diagnosis or intervention of primary focus followed by the words "case report"	2 (1)
Key Words	2	2 to 5 key words that identify diagnoses or interventions in this case report, including "case report"	54 (2)
Abstract (no references)	3a	Introduction: What is unique about this case and what does it add to the scientific literature?	16-18 (2)
	3b	Main symptoms and/or important clinical findings	23-26 (2), 35-38 (2)
	3c	The main diagnoses, therapeutic interventions, and outcomes.	27-29 (2), 39-42 (2)
	3d	Conclusion—What is the main "take-away" lesson(s) from this case?	45-50 (2)
Introduction	4	One or two paragraphs summarizing why this case is unique (**may include** references)	44-50, 19-26 (3)
Patient Information	5a	De-identified patient specific information	4-7 (5), 24-26 (6)
	5b	Primary concerns and symptoms of the patient	7-11 (5), 24-26 (6)
	5c	Medical, family, and psycho-social history including relevant genetic information	17-19 (5), 31-35 (6)
	5d	Relevant past interventions with outcomes	10-13 (5), 32-34 (6)
Clinical Findings	6	Describe significant physical examination (PE) and important clinical findings	23-30 (5), 40-47 (6)
Timeline	7	Historical and current information from this episode of care organized as a timeline	34-38 (5)
Diagnostic Assessment	8a	Diagnostic testing (such as PE, laboratory testing, imaging, surveys).	41-51 (5), 49-53 (6)
	8b	Diagnostic challenges (such as access to testing, financial, or cultural)	49-51 (5), 56-60 (6)
	8c	Diagnosis (including other diagnoses considered)	60-62 (5), 60-62 (6)
	8d	Prognosis (such as staging in oncology) where applicable	1-3 (6), 60-62 (6)
Therapeutic Intervention	9a	Types of therapeutic intervention (such as pharmacologic, surgical, preventive, self-care)	6-9 (6), 6-8 (7)
	9b	Administration of therapeutic intervention (such as dosage, strength, duration).	9-11 (6), 8-11 (7)
	9c	Changes in therapeutic intervention (with rationale)	10-11 (6), 11-13 (7)
Follow-up and Outcomes	10a	Clinician and patient-assessed outcomes (if available).	
	10b	Important follow-up diagnostic and other test results.	11-12 (6), 13-15 (7)
	10c	Intervention adherence and tolerability (How was this assessed?)	12 (6), 13-15 (7)
	10d	Adverse and unanticipated events.	10 (6)
Discussion	11a	A scientific discussion of the strengths AND limitations associated with this case report	23-29, 18-22 (9)
	11b	Discussion of the relevant medical literature **with references**	31-45 (9)
	11c	The scientific rationale for any conclusions (including assessment of possible causes).	58-61 (9)
	11d	The primary "take-away" lessons of this case report (without references) in a one paragraph conclusion	1-4, 6-8 (10)
Patient Perspective	12	The patient should share their perspective in one to two paragraphs on the treatment(s) they received	12-17, 24-28 (10)
Informed Consent	13	Did the patient give informed consent? Please provide if requested	**Yes** X **No** ☐
